# Burden and factors associated with onchocerciasis transmission among school-aged children after more than 20 years of Community Directed Treatment with Ivermectin in Ulanga district, Tanzania: A school-based cross-sectional study

**DOI:** 10.1371/journal.pgph.0001919

**Published:** 2023-05-12

**Authors:** Mwanahawa I. Mshana, Valeria Silvestri, Vivian Mushi, Witness M. Bonaventura, Donath Tarimo, Billy Ngasala, Dinah B. Gasarasi

**Affiliations:** 1 Department of Parasitology and Medical Entomology, School of Public Health and Social Sciences, Muhimbili University of Health and Allied Sciences, Dar es Salaam, Tanzania; 2 Department of Zoology and Wildlife Conservation, College of Natural and Applied Sciences, University of Dar es Salaam, Dar es Salaam, Tanzania; 3 Department of Medical Laboratory Science, Kilimanjaro Christian Medical University College, Kilimanjaro, Tanzania; Christian Medical College Vellore, INDIA

## Abstract

Onchocerciasis transmission in the Ulanga District of Morogoro-Tanzania is still ongoing despite more than 20 years of Community Directed Treatment with Ivermectin (CDTI) in the area. Even though surveys conducted over the years in the area have revealed a decrease in the prevalence of onchocerciasis, the prevalence of comorbidities suggested to be associated with this filarial infection, as epilepsy, is rising in endemic regions. This fact suggests continued transmission of *Onchocerca volvulus* and emphasizes the need for the evaluation of factors associated with it among school aged children. Therefore, this study determined the current burden of onchocerciasis in children aged 6 to 12 years and factors associated with continued transmission despite more than 20 years of CDTI in Ulanga District, Morogoro Region-Tanzania. A school-based cross-sectional study was conducted among 270 children aged 6 to 12 years in Ulanga District. Participants were tested using the OV-16 IgG4 Rapid Test. An interview-administered questionnaire was used to assess disease-associated symptoms, knowledge of onchocerciasis, and factors associated with continued transmission of the disease among participants. Descriptive statistics, chi-square test, and logistic regression were performed during data analysis. The prevalence of onchocerciasis was 19.6% (53/270), with boys being more infected; prevalence increased with increasing age groups and was higher in rural areas. Sex (AOR = 2.2, 95% CI: 1.13–4.28), age group of 11–12 years (AOR = 31.45, 95% CI: 2.73–362.27), and not taking ivermectin (AOR = 3.17, 95% CI: 1.53–6.58) were the only significant factors identified to be associated with the transmission of onchocerciasis in this study. The high prevalence of onchocerciasis among school age children in Ulanga district suggests continued transmission in the area. Therefore, a need to integrate CDTI with health education on the importance of ivermectin uptake.

## Introduction

Onchocerciasis is a neglected tropical disease caused by the filarial nematode *Onchocerca volvulus* (*O*. *volvulus*), which is endemic in African countries, with some foci in Latin America and Yemen [1]. Onchocerciasis is common in areas with fast-flowing rivers and streams, which are suitable breeding sites for black flies vectors (*Simulium* species) [1]. The disease is transmitted to humans by the bite of an infected female black fly. Onchocerciasis threatens the livelihood of individuals by incapacitating them and affecting work life, affecting growth and cognitive ability (Nakalanga syndrome), and eventually causing death [1].

According to the global burden of disease study estimate, there were 20.9 million *O*. *volvulus* infections worldwide in 2017; 14.6 million of the infected people had skin disease and 1.15 million had vision loss [2,3]. More than 99% of infected people live in 31 African countries [2]. Data from different studies and different regions may differ. Data from Sierra Leone registered a seroprevalence for *O*. *volvulus* exposure of 3.3%, with only a minority of positive cases aged less than 18 years [4], while an analysis of studies from different African regions reported a seroprevalence up to 89.3% observed in Democratic Republic of Congo among participants aged ≤10 years in 2014–2016 [5]. Onchocerciasis affects all age groups from children under ten years to adults above fifty years with the trend of seroprevalence varying across the age between different studies, in some of them higher seroprevalence was observed in older age compared to younger children, while in other studies a higher seroprevalence was observed in the younger children compared to the older [5].

The disease manifestations principally affect the skin tissue, with the dermal layer parasitized by the microfilaria stage. Symptoms, which include debilitating itching, loss of skin pigmentation which is common in adults, and groin enlargement are due to the intense inflammatory reaction secondary to the microfilaria death [4,6]. Furthermore, ocular lesions with keratitis and retinal and optic nerve involvement can result from the migration of microfilariae to eye tissues and the inflammatory response invoked by their death, leading to blindness commonly known as river blindness. Specific neurological Nodding syndrome and impairment of growth, called Nakalanga syndrome have also been reported in patients mainly children [5]. Even though the pathophysiological mechanisms still need to be elucidated, current epidemiological evidence indicates that there is an increased prevalence of epilepsy among children in onchocerciasis endemic regions suggesting that *O*. *volvulus* could be a trigger of epilepsy [4,7].

Over the years, onchocerciasis control targets shifted from morbidity control to disease elimination in endemic African countries, including Uganda, the Democratic Republic of Congo, Mali, and Tanzania under the World Health Organization (WHO) [7]. Treatment of individuals with ivermectin for 10 to 15 years through Mass Drug Administration (MDA**)** programs annually or biannually was suggested by the African Program for Onchocerciasis Control (APOC) as a means for disease elimination because the drug used, ivermectin, kills the microfilaria but only partly paralyzes the adult worm, and must be taken for the reproductive lifespan of the worm to counteract transmission [8]. The program (APOC) ended in 1995, and individual countries were responsible for ensuring disease elimination [9].

In Tanzania, 6 million people are at risk of acquiring onchocerciasis, dwelling in the endemic areas of Morogoro, Tanga, Iringa, Lindi, Mbeya, and Ruvuma. Morogoro was the first region to be identified as hyperendemic, specifically Ulanga district, which had 60% and 95% microfilaria and nodule prevalence, respectively, leading to the introduction of an onchocerciasis control program (CDTI) in 1997 in collaboration with the APOC [10,11]. CDTI is a community-based and operated strategy for ivermectin distribution from house to house using community drug distributors. The utilization of the CDTI program increases ownership of the program through decision-making regarding the need for treatment, logistics for distribution, and empowerment. For evaluation of the elimination of onchocerciasis transmission, the WHO requires 12 to 15 years of CDTI [12] which has already been achieved in the Mahenge-Ulanga foci in Morogoro, Tanzania [10,11]. Despite more than 20 years of annual CDTI, persistent transmission has been documented as being associated with poor compliance/acceptability of ivermectin treatment, inadequate knowledge, negative attitudes and perceptions towards the CDTI program, and suboptimal coverage of the CDTI program [10,11]. Data from Mahenge reported a seroprevalence of up to 48.1% among children aged 9 years in 2018 [13]. The persistent transmission of onchocerciasis led to the introduction of the bi-annual CDTI in 2019 to accelerate disease control.

The 2019–2020 WHO’s progress report on elimination of human onchocerciasis recommends that mapping should target areas of greatest concern and identify areas in which there is no risk of transmission [14]. The previously reported ongoing transmission of onchocerciasis in the Mahenge foci [5] suggests the need for reassessment in this area. Several factors may have a role in the ongoing transmission [10,11] and have to be additionally described. Along with the need for strengthening control and elimination strategies, it should be considered that MDA alone could be insufficient in reaching the goals, hence the need for vector control interventions [15]. This study was therefore designed to assess current situation of onchocerciasis among school aged children, a population that according to WHO guidelines is the best to assess the ongoing transmission of the disease [14]. The information acquired will guide programs aimed at elimination of onchocerciasis in accordance to 2021–2030 WHO NTD road map [16].

## Materials and methods

### Ethics statement

Ethical clearance was obtained from the institutional review board of Muhimbili University of Health and Allied Sciences (MUHAS-REC-05-2022-1181). Permission from local authorities, including the Regional Administrative Secretary of Morogoro and the District Administrative Secretary of Ulanga was acquired. Confidentiality was ensured, the head teacher signed consent forms on behalf of the children’s parents who were previously informed and verbally agreed. The procedures followed were per the ethical standards of the Helsinki Declaration (1964, amended most recently in 2008) of the World Medical Association.

### Study setting

The study was conducted in the mountainous area of Ulanga district of Morogoro Region, South Eastern Tanzania. With an elevation of 3408 feet above sea level, the area covers 2,802.29 square kilometers and is surrounded by rivers Luli, Mbalu, Lukande, Mzelezi, Ruaha, and Msingizi, which serve as the main breeding sites for the black fly vectors whose infective bites transmit onchocerciasis. Miombo forests and woodlands additionally favor the survival of black flies. The area is characterized by a rainy season that occurs from November to May and a dry season from June to October. Ulanga district has a total population of 165,903 people [82,784 males, 83,119 females] [17]. Individuals are mainly engaged in small scale agricultural activities (livestock keeping and crop farming) and small scale mining. The district was chosen because of its history of onchocerciasis transmission dating back to 1960 and its persistence despite more than 20 years of CDTI. The available data reported a seroprevalence of up to 48.1% among children aged nine years in 2018 [10,11,13,18].

### Study design, participants, and inclusion and exclusion criteria

A primary school-based cross-sectional study was conducted in Ulanga District, Morogoro region, Tanzania, between June and July 2022 to investigate the current burden and factors associated with the transmission of onchocerciasis among school-aged children.

The inclusion criteria were the age of 6–12 years old from class 1 to 5. WHO recommends including a pediatric population aged <10 years, suggesting it’s the best for assessing active transmission of infection. The participants whose parents verbally agreed and were willing to participate were considered for inclusion in the study, while the exclusion criterion was children unable to communicate due to medical conditions.

### Sample size estimation and sampling technique

The sample size was determined using a formula for the binary outcome (positivity for previous *O*. *volvulus* exposure on rapid antigen test) in a cross-sectional study design, developed by Leslie Kish: n = z^2^ p (1-p)/ℇ^2^ (n = sample size, z = standard normal deviate (1.96) on using 95% CI, p = expected prevalence of onchocerciasis (33.6%), ℇ = margin of error (5%) [20]. Considering the non-response rate (10%), the estimated sample size was 376 school-aged children. However, because the school had previously scheduled a trip for the classes on the days of the fieldwork, cutting into the time for assessment, only 270 school-aged children were enrolled. The sample size reduction occurred randomly.

**Fig 1:**
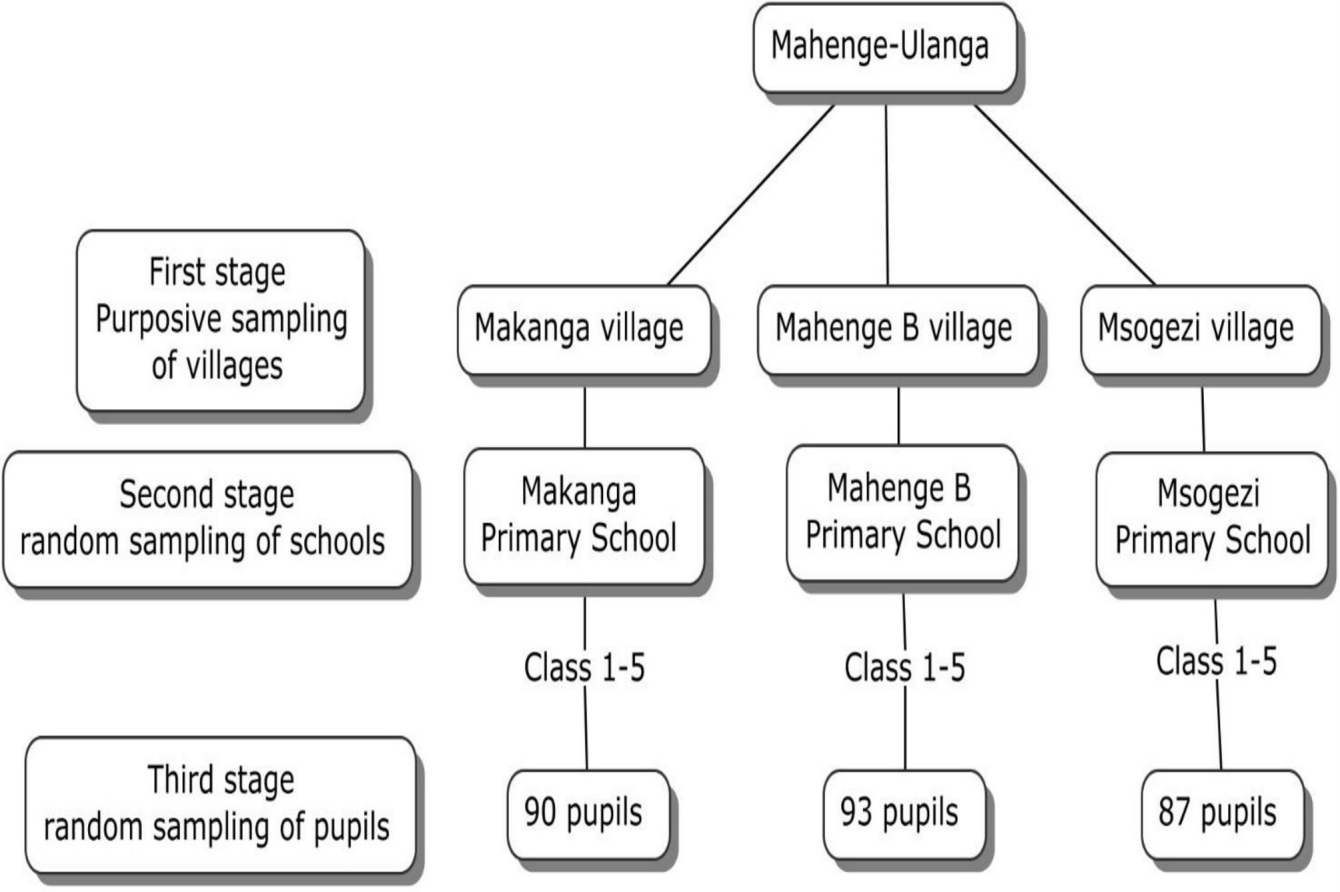
Flow chart of sampling technique.

A sampling of the study participants was done using cluster sampling (Fig 1). In the first stage, among all villages in the Ulanga district, three of them were purposely selected according to known previous endemicity: Mahenge B, Makanga, and Msogezi. In the second stage, the total number of schools in the selected villages was determined, and one school was selected from each village at random. In the third stage, simple random sampling was used to select students from each school. A total of 270 children aged 6 to 12 (class 1–5) years residing in Mahenge B, Makanga, and Msogezi villages of the Vigoi Division in Ulanga were included. Children in class 1–5 included children aged 6–12, which was conveniently chosen in order not to exclude participants from a selected class because of slight overage if referring to WHO guidelines suggesting a population <10 years of age to document interruption of transmission [18]

### Data collection methods and tools

#### Structured interview using a questionnaire

A pre-designed interviewer administered questionnaire was used to determine; anagraphical and anamnestic data, the knowledge on onchocerciasis and the practices that favored transmission of onchocerciasis among participants in the study area. The tool was written self constructed in English then translated and administered in Kiswahili by the bilingual principal investigator and cross checked by the supervisors, to address the study aims and objectives. The questionnaire was administered by bilingual researchers mastering the field of study. The foreigner research assistant had certificated knowledge of local language and was additionally trained before implementation of the project in the field. Questionnaires (English and Kiswahili versions) are attached as S1 and S2 Texts. For the questions analyzing skin changes, any positive reports from the respondents were checked clinically and confirmed by the interviewer. For epilepsy and seizures, the interviewer made sure the definition of the condition was clear to the respondent, describing the disease in simple words and with examples. The same was done for the question on ivermectin uptake to reduce the bias due to the limitation of a word-of-mouth assessment.

#### Diagnostic tools

To determine the prevalence of previous exposure to *O*. *volvulus*, participants’ finger capillary blood was tested using OV-16 IgG4, SD bioline *onchocerca* rapid test kits (Abbot Diagnostics Inc, Giheung-gu, Yongin-si, Gyeonggi-do, 17099, Republic of Korea) which detects the presence of antibodies towards *Onchocerca volvulus* antigens with a sensitivity of 81.1% and specificity of 99%.

#### Data management and analysis

Data entry into a standardized questionnaire was done using the REDCap electronic data capture tool hosted at Kilimanjaro Christian Medical University College and was later transferred to Excel (S1 Data) and cleaned. Data protection was ensured by anonymization before using analytical software. Data was analyzed using the STATA software for Windows version 14.0 (StataCorp. 2015. Stata Statistical Software: Release 14. Texas 77845 USA).

Independent and dependent variables were summarized using descriptive statistics, which were reported as frequencies and proportions. The chi-square test was used to compare categorical variables with a significance level of p < 0.05. To identify the variables with p < 0.25 to include in the multivariate logistic regression, a univariate logistic regression analysis was performed. Factors and practices associated with onchocerciasis were determined using multivariate analysis. The dependent variable in the logistic regression analysis was the rapid test positivity defining *O*. *volvulus* previous exposure. The results were reported as COR and AOR with a 95% confidence interval.

## Results

### Socio-demographic characteristics of the study participants

A total of 270 pupils were included in this study, of which more than half were girls (158/270, 58.5%). The age range was 6–12 years, and the majority (94.4%, 255/270) had lived in the area for >5 years (Table 1).

**Table 1:** Demographic characteristics of study participants (n = 270).

Variable	Frequency (%)
**Sex**	
Boy	112(41.5)
Girl	158 (58.5)
**Age group**	
6–8	78 (28.9)
9–10	113 (41.8)
11–12	79 (29.3)
**Class**	
Class 1	34 (12.6)
Class 2	69 (25.6)
Class 3	47 (17.4)
Class 4	63 (23.3)
Class 5	57 (21.1)
**Village name**	
Makanga	90 (33.3)
Mahenge	93 (34.5)
Msogezi	87 (32.2)
**Duration of residency**	
< 5 Years	15 (5.6)
> 5 Years	255 (94.4)
**Location of residence**	
With rivers	177 (65.8)
Without rivers	92 (34.2)

### Symptoms of onchocerciasis among the study participants

When analyzing the prevalence in our study participants of signs and symptoms known to occur among individuals infected with onchocerciasis, including itching, nodules on the body, eye redness or discomfort, skin changes, and seizures, we observed that periodic eye redness and discomfort was the most reported symptom (25.9%, 70/270). Participants that never reported periodic eye redness had the highest percentage of OV16 positivity (21.5%, 43/200). None of the signs and symptoms assessed was significantly associated with a positive result to OV16 test (Table 2).

**Table 2:** Signs and symptoms of onchocerciasis and infection status (n = 270).

Signs and symptoms of onchocerciasis	Total Number	Infection status		p-value
		Positive	Negative	
**Experience itching**				
Always	4	0 (0.0)	4 (100)	0.140
Periodically	64	8(12.5)	56 (87.5)	
Never	202	45 (22.3)	157(77.7)	
**Nodules on the body**	0	53 (19.6)	217 (80.4)	NA
**Eye redness or discomfort**				
Always	6	0 (0.0)	6 (100)	0.278
Periodically	64	10 (15.6)	54 (84.4)	
Never	200	43 (21.5)	157 (78.5)	
**Skin changes**				
Skin desquamation	0	0 (0.0)	0 (0.0)	0.782
Leopard skin	2	0 (0.0)	2 (100)	
Loss of skin elasticity	5	1 (20.0)	4 (80.0)	
No changes	263	52 (19.8)	211 (80.2)	
**Experienced seizures**				
Yes, I was told	2	1 (50.0)	1 (50.0)	0.548
I don’t know	147	28 (19.1)	119 (80.9)	
I have never	121	24 (19.8)	97 (80.2)	

*Statistically significant (p< 0.05).

### Prevalence of onchocerciasis (based on IgG4- OV16 RDT) among the study participants

The prevalence of onchocerciasis was 19.6% (53/270). Prevalence varied according to sex and increased significantly with age, with the highest prevalence being 23.2% (26/79) in the age group 11–12 years. In addition, the high prevalence of onchocerciasis was observed among boys aged 11–12 years (16/37, 43.2%, p = 0.001) and girls aged 11–12 years (10/42, 23.8%, p = 0.003) when compared to rest of age groups (S1 Table). Prevalence was also higher in the rural (37.9%, 33/87) compared to suburban and urban areas (Table 3). No statistically significant difference was observed among participants living near or far from the river.

**Table 3:** Prevalence of onchocerciasis according to socio-demographic characteristics of study participants (n = 270).

Variable	Total Number	Infection status		p-value (chi-square test)
		Positive	Negative	
**Sex**				
Boy	112	29 (25.9)	83 (74.1)	0.029*
Girl	158	24 (15.2)	134 (84.8)	
**Age group**				
6–8	78	1 (1.3)	77 (98.7)	0.001*
9–10	113	26(23.0)	87 (77.0)	
11–12	79	26 (23.2)	53 (67.1)	
**Class**				
Class 1	34	1 (2.9)	33 (97.1)	0.001*
Class 2	69	5 (7.2)	64 (92.8)	
Class 3	47	11 (23.4)	36 (76.6)	
Class 4	63	17 (27.0)	46 (73.0)	
Class 5	57	19 (33.3)	38 (66.7)	
**Village name**				
Makanga (sub-urban)	90	17 (18.9)	73 (81.1)	0.001*
Mahenge (urban)	93	3 (3.2)	90 (96.8)	
Msogezi (rural)	87	33 (37.9)	54 (62.1)	
**Duration of stay**				
< 5 Years	15	0 (0.0)	15 (100)	0.049*
>5Years	255	53 (20.8)	202 (79.2)	
**Location of residence**				
With rivers	177	38 (21.5)	139 (78.5)	0.056
Without rivers	93	15 (16.1)	78 (83.9)	

*Statistically significant (p< 0.05).

### Practices associated with the transmission of onchocerciasis among the study participants

#### Activities in the forest and around rivers

Of the 270 participants, 34/270 (12.6%) reported going to the forest and rivers for activities; such as hunting (2/34, 5.9%), fruit picking (2/43, 5.9%), playing (6/34, 17.6%), and other activities, such as firewood collection, fetching water, farming (24/34, 70.6%). Of the 34 participants who had a practice of going to the forest and rivers, 7 (20.6%) were onchocerciasis infected. However, none of the activities in the forests and around rivers that usually can expose individuals to blackflies was significantly associated with onchocerciasis.

#### Water sources used by the participants

More than one-third of the participants (94/270, 34.8%) reported using water from three sources (taps, wells, rivers), followed by those who use water from the tap (65/270, 24.1%), rivers (43/270, 15.9%), taps and rivers (25/270, 9.3%), taps and wells (22/270, 8.1%) and wells (21/270, 7.8%).

A higher prevalence of onchocerciasis was observed among the participants who used water from the rivers (13/43, 30.2%), followed by those who reported using water from wells (5/21, 23.8%), water from three sources (taps, well, river) (20/94, 21.3%), tap and river (5/25, 20.1%), taps and wells (3/22, 13.6%) and taps only (7/65, 10.8%). None of the water sources that usually expose individuals to blackflies was significantly associated with onchocerciasis (p = 0.206).

#### Ivermectin uptake among the study participants

Of the 270 participants, more than 51.9% (140/270) reported not taking ivermectin in the last round of distribution with no reasons for not taking the drugs (94.2%, 132/140). When compared to other age groups, the 9–10 year group had significantly higher ivermectin uptake (61/130, 46.9%) and onchocerciasis prevalence (19/61, 31.1%, p = 0.026). A significant association was found between taking ivermectin in the last round of distribution and positive infection status (p = 0.001) (Table 4). In addition, ivermectin uptake was associated with age groups (p = 0.001), student classes (p = 0.001), villages of residents (p = 0.001), and duration of the residency (p = 0.025) (S2 Table).

**Table 4:** Ivermectin uptake and infection status (n = 270).

Ivermectin uptake	Total	Infection status		p-value
		Positive	Negative	
**Ivermectin uptake in the last round**				
Yes	130	37 (28.5)	93 (71.5)	0.001*
No	140	16 (11.4)	124 (88.6)	
**Reasons for not taking medication**				
Side effects of the drug	1	0 (0.0)	1 (100)	0.026*
Parent’s restriction	7	3 (42.9)	4 (57.1)	
I don’t know	132	13 (9.8)	119 (90.2)	
**Ivermectin uptake according to the participants sex**				
**Boy**				
Yes	59	23 (39.0)	36 (61.0)	0.000*
No	53	06(22.3)	47 (88.7)	
**Girl**				
Yes	71	14 (19.7)	57 (80.3)	0.152
No	87	10 (11.5)	77 (88.5)	
**Ivermectin uptake according to age groups**				
6–8 years				
Yes	12	00 (0.0)	12 (100)	0.668
No	66	01 (1.5)	65 (98.5)	
9–10 years				
Yes	61	19 (31.1)	42 (68.9)	0.026*
No	52	07 (13.5)	45 (86.5)	
11–12 years				
Yes	57	18 (31.6)	39 (68.4)	0.685
No	22	08 (36.4)	14 (63.6)	

*Statistically significant (p< 0.05).

#### Factors associated with continued onchocerciasis transmission among the study participants

The factors significantly associated with continued onchocerciasis transmission were being male (AOR = 2.2 95% CI 1.13–4.28), age group of 11–12 years (AOR = 31.45 95% CI 2.73–362.27), and not taking ivermectin in the last round of distribution (AOR = 3.17 95% CI 1.53–6.58) (Table 5).

**Table 5:** Factors associated with continued transmission of onchocerciasis among the study participants.

Variable	Univariate analysis		Multivariate analysis	
	Crude Odds Ratio	p- value	Adjusted Odds Ratio	p -value
**Sex**				
Girl	Ref.			
Boy	1.95 (1.06–3.58)	0.031*	2.2 (1.13–4.28)	0.021*
**Age**				
6–8	Ref.			
9–10	23.01 (3.05–173.59)	0.002*	21.55 (2.01–230.63)	0.011*
11–12	37.77 (4.97–286.96)	0.001*	31.45 (2.73–362.27)	0.006*
**Tap water**				
Yes	Ref.			
No	1.99 (1.07–3.72)	0.031*	1.58 (0.78–3.20)	0.201
**Ivermectin uptake in the last distribution round**				
Yes	Ref.			
No	3.08 (1.61–5.88)	0.001*	3.17 (1.53–6.58)	0.002*

*Statistically significant (p< 0.05).

## Discussion

This study was designed to assess the current burden of onchocerciasis and factors associated with continued transmission among school-aged children after more than twenty years of CDTI in Ulanga District, Tanzania. The presence of disease (based on antibody detection) in children represents exposure to *O*. *volvulus* among this young age group, which is indicative of continued transmission despite the treatment efforts (CDTI), put in place for parasite control and elimination of onchocerciasis.

A high prevalence of onchocerciasis was observed in the study area (19.6%) much higher, compared to the WHO guidelines that require a minimum prevalence of 0.1% to consider elimination [9,12]. When comparing this finding to recent data from different African nations, we can observe that different from what was reported in studies from Nigeria, and from some regions in DRC, where no positivity was reported in children under 10 years old participating in the study, there is still need to put the effort in the implementation of the prevention and control measures in our setting [5]. The prevalence reported in our setting is low compared to other settings, like South Sudan, where a prevalence of 33.3% was reported in 2020 or the overall prevalence of 55.8% reported in Cameroon in 2017 [5]. When analyzing the prevalence in the country of Tanzania, the situation in Mahenge is improved when compared to the prevalence reported for rural areas in the region in 2018, which was 38.3% [5].

The prevalence was significantly higher in boys compared to girls and was associated with older age groups. A recent study analysing human antibody levels to *Onchocerca’s* vector *Simulium damnosum* s.l. saliva in endemic areas observed a lower IgG response in males than females. This was attributed to immune tolerance and desensitization with cumulative saliva exposure in males. It has been suggested that there are sex differences in behaviour, such as daily habits, occupation, education, or clothing, that influence physical exposure to blood-seeking blackflies, which are more prevalent in male. In addition, it has been suggested that girls show greater levels of non-specific innate and adaptive immune responsiveness than boys, particularly post-puberty, and that hormonal involvement may contribute to the observed sex differences [19].

The onchocerciasis prevalence also followed a pattern of the area; it was higher in the rural locations compared to the urban areas. This difference could be explained by the presence of forest and the common use of river water among individuals living in rural areas as opposed to those in the urbanized sites where vegetation cover is cleared for infrastructure development and the use of tapped water supplies instead of going to the river water source, thus reducing the risk of coming into contact with the black fly vectors (*Simulium* species). This study reports similar findings to those conducted in Mahenge; an onchocerciasis seroprevalence of 20.7% was observed in Mahenge among children aged 6–10 years, with a pattern of prevalence similar to the one observed in our study according to the area (urban and rural) and age but no significant difference in prevalence according to sex in both settings [13]. The increasing seroprevalence of onchocerciasis according to age was also reported in other studies from African countries, which confirm the cumulative nature of the disease, having a higher prevalence in older children and rural compared to the urbanized areas [20,21]. Even though discordant findings are reported about gender differences in distribution of seroprevalence, a study in Cameroon has also observed lower infection status among girls [22,23], as we did in our study.

Of the symptoms described to occur in people infected with *O*. *volvulus*, periodic itching, skin changes, eye discomfort/visual impairment, and seizures were reported by participants included in this study. Symptoms and signs are not pathognomonic, and while some of these symptoms were evident among those identified to have been exposed to *O*. *volvulus*, some of the participants who tested negative for onchocerciasis reported similar or had similar visible symptoms which might have been caused by different etiologies such as bacterial and fungal infections or might be due to false negative results. The study was not powered to rule out differential diagnosis. No significant association was found between these symptoms and onchocerciasis. Several factors can explain the mismatch between clinical presentation and seroprevalence. One of these could be the younger age of the participants since the symptoms manifest later in life due to the chronic nature of the disease [21] and the other could be due to the extensive treatment (25 years) with ivermectin in the area that has lowered the microfilaria load associated with the development of symptoms in onchocerciasis infected individuals.

No nodules were observed among the study participants confirming results to a study in Cameroon where results showed recent ivermectin treatment had an association with lower nodule prevalence [23]. The lack of nodules may be explained by the long time needed for the adult worm to fully develop forming nodules or by the arrest of the growth of microfilaria into adult worms due to ivermectin. A study assessing epilepsy in onchocerciasis endemic areas showed high IgG4-OV16 prevalence in individuals with confirmed epilepsy compared to those without epilepsy [24]. In our study we found, there was no statistically significant difference in onchocerciasis prevalence between the participants who had experienced seizures before and those who didn’t know if they had ever experienced seizures. This might be explained by the fact that in the current study the sample size was limited compared to the earlier study. Secondly, the use of reported epilepsy cases instead of using a confirmed clinical diagnosis may also have contributed to the indicated differences between the two studies, raising concerns about potential reporting bias. It is of interest that low epilepsy prevalence, as in the current study, was reported in a study done in Nigeria after more than 20 years of CDTI [25], which shows similarity to the results in the current study, due to more than 20 years of CDTI in the study area (Ulanga District).

No ocular lesions or visual impairment was reported among our participants, different to what was observed by other authors in different contexts. A study in Central and Northern Togo revealed presence of ocular lesions due to onchocerciasis in children and young adults during ophthalmological assessment [26]. As for the neurological conditions, the lack of physical and instrumental assessment could have led to underreporting bias in our setting. The absence of clear symptoms of onchocerciasis among school aged children in the current study area could also be due to continued treatment with ivermectin lowering the microfilaria load hence limiting the pathological effects that lead to the development of the symptoms.

Activities in the forests and around rivers that expose individuals to the black flies propagate continued transmission; in this study, none of these activities were significantly associated with onchocerciasis. Proximity to rivers breeding sites for the vector have been described to influence the continued transmission of onchocerciasis by increasing exposure. The annual or bi-annual ivermectin uptake reduces the microfilaria load in individuals and thus is likely to have controlled transmission. Poor compliance with ivermectin treatment has been identified as a practice associated with onchocerciasis transmission. In this study, the participants who did not take ivermectin were three times at higher risk of acquiring onchocerciasis compared to those who took ivermectin, hence, being a source of continued transmission of onchocerciasis due to harboring an uncontrolled microfilaria load. Previous studies conducted in other settings suggested that in areas with low CDTI coverage transmission was still high compared to those with high coverage of CDTI, aligning with the results of the current findings, which confirm that ivermectin coverage and uptake have an association with continued onchocerciasis transmission [25]. In Cameroon, a comparison between pre CDTI and post CDTI established and proved a significant decrease in the circulating microfilaria load among communities involved in the study showing a direct link between ivermectin uptake and the transmission of onchocerciasis [23]. Another study in Togo also revealed lowered onchocerciasis prevalence after MDA with ivermectin translating the direct effect of ivermectin uptake on transmission of onchocerciasis [26]. Lower epilepsy prevalence was observed in a study done in Nigeria showing minimal transmission as a result of more than 20 years of CDTI [25], also suggesting a potential effect of ivermectin uptake on the reduction of morbidity observed in endemic areas for onchocerciasis. The high number (140) of individuals who reported not taking ivermectin in the current study were mostly in the lower classes (1 and 2); this could be due to being excluded in previous MDA programs because of younger age.

Transmission is also affected by self protection behavior taken by individuals against the black fly bites; participants in this study reported putting on protective clothing, including trousers and long sleeved shirts to prevent being bitten by black flies, when going to the farm, a habit which could prevent transmission. It has been suggested that women’s clothes may cover more, leaving fewer regions exposed to vector bite, thus contributing to justify the gender differences in the prevalence of positivity for *Onchocerca* exposure [20].

### Study limitations

The limited self-explanatory ability among children makes this group more prone to biased answers. Also, random sampling was carried out among school children. This could have left out any participant that, because of severe morbidity due to the disease, had dropped out of school. A physical examination of all participants that could have made the report of symptoms and signs more accurate was not included in the project activity in this preliminary study, leading to a potential underreporting of findings. The information on the ivermectin uptake was collected only by word of mouth hence could be subjected to recall bias and could result in over or underestimation of the prevalence of ivermectin uptake. Due to time constraints during fieldwork, the sample size was reduced from the original estimate; this happened randomly, but it is still possible that this had a significant impact on the findings. In addition, the study could not account for false positives and false negative tests because we did not conduct a confirmatory test. Further studies are needed to address this limitation and describe the clinical conditions of school-aged children in this setting.

## Conclusions and recommendations

A high prevalence (19.6%) of onchocerciasis has been established among school-aged children in the Ulanga district despite more than 20 years of CDTI, suggesting continued transmission in the area. Factors that were associated with onchocerciasis transmission in this study were age, sex, and ivermectin uptake. While CDTI is being implemented, there is also a need to integrate it with health education on the importance of ivermectin uptake since individuals that are non-adherent to CDTI act as reservoirs for continued transmission.

## Supporting information

S1 Table Prevalence of onchocerciasis across the age groups by sex. (DOCX)

S2 Table Prevalence of ivermectin uptake according to the socio-demographic characteristics of the participants. (DOCX)

S1 Text This is the questionnaire in English. (DOCX)

S2 Text This is the questionnaire in Kiswahili. (DOCX)

S1 Data The data set used for analysis. (CSV)
