# Supplementary material for: Burden and factors associated with onchocerciasis transmission among school-aged children after more than 20 years of Community Directed Treatment with Ivermectin in Ulanga district, Tanzania: A school-based cross-sectional study
Source: PLOS Glob Public Health. 2023 May 12;3(5):e0001919. doi: 10.1371/journal.pgph.0001919 (PMC10180657; doi:10.1371/journal.pgph.0001919)
Supplement: S1 Table — (DOCX) [file pgph.0001919.s001.docx]

| **Age group** | **Sex** | | | | | |
| --- | --- | --- | --- | --- | --- | --- |
|  | **Male** | | | **Female** | | |
|  | **Positive** | **Negative** | **p-value** | **Positive** | **Negative** | **p-value** |
| 6-8 | 01 (3.0) | 32 (97.0) | 0.001* | 00 (0.0) | 45 (100) | 0.003* |
| 9-10 | 12 (28.6) | 30 (71.4) |  | 14 (19.7) | 57 (80.3) |  |
| 11-12 | 16 (43.2) | 21 (56.8) |  | 10 (23.8) | 32 (76.2) |  |

**S1 Table. Prevalence of onchocerciasis across the age groups by sex**
