# Supplementary material for: Burden and factors associated with onchocerciasis transmission among school-aged children after more than 20 years of Community Directed Treatment with Ivermectin in Ulanga district, Tanzania: A school-based cross-sectional study
Source: PLOS Glob Public Health. 2023 May 12;3(5):e0001919. doi: 10.1371/journal.pgph.0001919 (PMC10180657; doi:10.1371/journal.pgph.0001919)
Supplement: S2 Table — (DOCX) [file pgph.0001919.s002.docx]

| **Demographic Variable** | **Total** | **Ivermectin use** | | **p-value** |
| --- | --- | --- | --- | --- |
|  |  | **Yes** | **No** |  |
| **Sex** |  |  |  |  |
| Male | 112 | 59 (45.4) | 53 (37.9) | 0.210 |
| Female | 158 | 71 (54.6) | 87 (62.1) |  |
| **Age group** |  |  |  |  |
| 6-8 | 78 | 12 (9.2) | 66 (47.1) | 0.001* |
| 9-10 | 113 | 61 (46.9) | 52 (37.1) |  |
| 11-12 | 79 | 57 (43.9) | 22 (15.7) |  |
| **Class** |  |  |  |  |
| Class 1 | 34 | 3 (2.3) | 31 (22.1) | 0.001* |
| Class 2 | 69 | 17 (13.1) | 52 (37.1) |  |
| Class 3 | 47 | 26 (20.0) | 21 (15.0) |  |
| Class 4 | 63 | 45 (34.6) | 18 (12.9) |  |
| Class 5 | 57 | 39 (30.0) | 18 (12.9) |  |
| **Village name** |  |  |  |  |
| Makanga | 90 | 32 (24.6) | 58 (41.4) | 0.001* |
| Mahenge | 93 | 28 (21.5) | 65 (46.4) |  |
| Msogezi | 87 | 70 (53.9) | 17 (12.2) |  |
| **Duration of resident** |  |  |  |  |
| <=5 Years | 15 | 3 (2.3) | 12 (8.6) | 0.025* |
| >5 Years | 255 | 127 (97.7) | 128 (91.4) |  |
| **Location of residence** |  |  |  |  |
| Near the river | 177 | 91 (70.5) | 86 (61.4) | 0.115 |
| Far from river | 93 | 38 (29.5) | 54 (38.6) |  |

**S2 Table. Prevalence of ivermectin uptake according to the socio-demographic characteristics of the participants**
