# Supplementary material for: Burden and factors associated with onchocerciasis transmission among school-aged children after more than 20 years of Community Directed Treatment with Ivermectin in Ulanga district, Tanzania: A school-based cross-sectional study
Source: PLOS Glob Public Health. 2023 May 12;3(5):e0001919. doi: 10.1371/journal.pgph.0001919 (PMC10180657; doi:10.1371/journal.pgph.0001919)
Supplement: S1 Text — (DOCX) [file pgph.0001919.s003.docx]

## S1: Questionnaire: English version

**
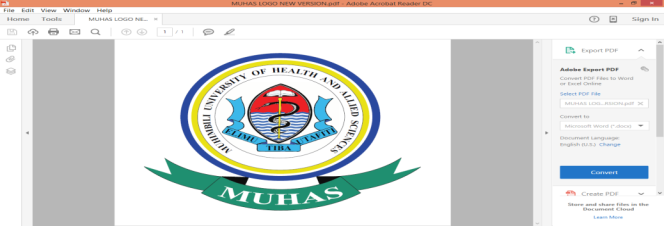
**

**MUHIMBILI UNIVERSITY OF HEALTH AND ALLIED HEALTH SCIENCES**

**DIRECTORATE OF RESEARCH AND PUBLICATIONS**

Hello, greetings! I am Mwanahawa Mshana a student from the Muhimbili University of Health and allied sciences. This is a form/ questionnaire intending to collect data for the research titled” Burden of Onchocerciasis among school aged children and factors associated with transmission after 20 years of community directed treatment with ivermectin in Mahenge town Ulanga district, Tanzania.” Information given will be handled with confidentiality and will be used only for the purpose stated above, no names will be recorded but identification numbers will be used instead. Thank you for your participation.

**ID**

**Put a tick (√) or an answer where appropriate.**

| **Section A:Sociodemographic characteristics** | | |
| --- | --- | --- |
| **SN.** | **Questions** | **Responses** |
| **1** | What is your age? |  |
| **2.** | Sex | 1. Male 2. Female |
| **3.** | What class are you in? |  |
| **4.** | How long have you lived in your current area of residence? | 1. Less than 5 years 2. More than 5 years 3. Lived here since I was born |
| **5.** | Where do you live? | 1. Close to the river(within 1500metres) 2. Away from the river(more than 1500 meters from the river) 3. Area without rivers |
| Section B: Assessment of signs and symptoms of Onchocerciasis | | |
| **6.** | Do you experience itching?  (pruritus) | 1. Always 2. Periodically 3. Never |
| **7.** | Have you got nodules on your body? | 1. Yes, on the hip bone 2. Yes, on the wrists 3. No, i don’t |
| **8.** | Do you experience eye redness or discomfort? | 1. Yes, always 2. Periodically 3. Never |
| **9.** | Do you experience any skin changes? | 1. Skin desquamation 2. Leopard skin 3. Loss of skin elasticity 4. No changes |
| **10.** | Have you ever experienced seizures? | 1. Yes, I was told 2. I don’t know 3. I have never |
| Section C:Practices associated with continued transmission of Onchocerciasis | | |
| **11.** | Do you perform any of these activities near or around rivers?  (tick where appropriate) | 1. Swimming 2. Fishing 3. Farming 4. Animal grazing 5. Bathing and washing 6. Only fetch water 7. No, i don’t |
| **12.** | Do you normally go to the forests? | 1. Yes 2. No |
| **13.** | If answered yes to question 21, what are the activities you normally do?  (tick where appropriate) | 1. Hunting 2. Fruit picking 3. Playing 4. Others |
| **14.** | If answered D. Others in question 22 , could you please list some of the activities |  |
| **15.** | Do you take ivermectin? | 1. Yes 2. No |
| **16.** | If not, what are the reasons? | 1. Side effects such as itching and body swelling 2. Distance travelled to acquire the drug 3. Parent’s restriction 4. I don’t know about the taking of the drug |
| **17.** | Do you take any preventive measures to protect yourself from being bitten by insects? | 1. Putting on long sleeved clothing 2. Putting on repellant 3. Others 4. I do not take any measures |
| **18.** | If answered C. others in question 25, can you give some of the preventive measures you take against being bitten by insects during the day |  |
| **19.** | Where do you normally get water from? | 1. Tap 2. Wells 3. River |
| **20.** | Laboratory results (OV16) | -Positive  -Negative |
