# Supplementary material for: Burden and factors associated with onchocerciasis transmission among school-aged children after more than 20 years of Community Directed Treatment with Ivermectin in Ulanga district, Tanzania: A school-based cross-sectional study
Source: PLOS Glob Public Health. 2023 May 12;3(5):e0001919. doi: 10.1371/journal.pgph.0001919 (PMC10180657; doi:10.1371/journal.pgph.0001919)
Supplement: S2 Text — (DOCX) [file pgph.0001919.s004.docx]

## S2:Questionnaire: Kiswahili version

**
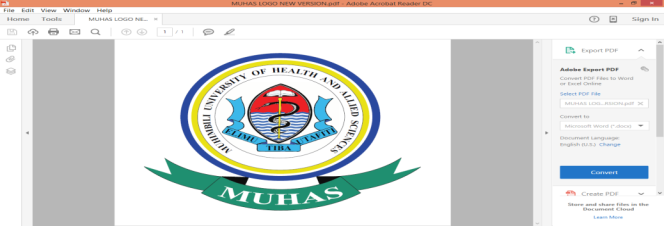
**

**CHUO KIKUU CHA AFYA NA SAYANSI SHIRIKISHI MUHIMBILI**

**KURUGENZI YA TAFITI NA MACHAPISHO**

Habari, ninatumai hujambo! Jina langu ni Mwanahawa Mshana, mwanafunzi kutoka chuo kikuu cha afya na sayansi shirikishi Muhimbili. Hii ni fomu/ dodoso yenye lengo la kukusanaya taarifa za utafiti wenye kichwa cha habari” Ugonjwa wa Usubi miongoni mwa watoto wenye umri wa shule na sababu zinazochangia kuendelea kusambazwa kwa ugonjwa baada ya miaka 20 ya matibabu kwa dawa ya Ivermectin katika mji wa Mahenge, wilaya ya Ulanga , Tanzania.” Taarifa zitakazokusanywa zitatumika kwa lengo la utafiti tajwa hapo juu tu na zitawekwa kwa usiri , majina hayatatumika katika kukusanya taarifa hizi ila namba za utambulisho.

Asante kwa kushiriki.

**NA.**

**Weka alama ya vyema (√) au jibu panapostahiki.**

| **Sehemu A:Utambulisho na taarifa za mshiriki** | | |
| --- | --- | --- |
| **NA.** | **Maswali** | **Majibu** |
| **1** | Una umri gani? |  |
| **2.** | Jinsia | 1. Me(mvulana) 2. Ke(msichana) |
| **3.** | Unasosma darasa la ngapi? |  |
| **4.** | Umeishi kwa muda gani katika eneo unaloishi sasa? | 1. Chini ya miaka 2 2. Zaidi ya miaka 2 3. Nimeishi hapa tangu nimezaliwa |
| **5.** | Unaishi wapi? | 1. Karibu na mto(ndani ya mita 1500) 2. Mbali na mto(zaidi ya mita 1500 kutoka kwenye mto) 3. Eneo lisilokuwa na mito |
| Sehemu B: Uchunguzi na Uangalizi wa dalili za ugonjwa wa Usubi | | |
| **6.** | Huwa unapata miwasho?  (pruritus) | 1. Mara zote 2. Mara chache 3. hapana |
| **7.** | Je, una uvimbe sehemu yoyote ya mwili wako? | 1. Ndiyo, kwenye mfupa wa nyonga 2. Ndiyo, kwenye viganja vya mkono 3. Hapana |
| **8.** | Je, huwa unapata maumivu au wekundu kwenye macho? | 1. Ndiyo, kila mara 2. Kwa vipindi , siyo mara zote 3. Hapana |
| **9.** | Umepata mabadiliko yoyote katika ngozi yako? | 1. Ngozi imekuwa ngumu 2. Ngozi imepata madoa madoa kama chui 3. Ngozi imepoteza asili yake ya kuvutika 4. Hapana sijapata mabadiliko yoyote |
| **10.** | Umewahi kupata kifafa? | 1. Ndiyo , niliambiwa 2. Sijui 3. Sijawahi |
| Sehemu C:Shughuli zinazochangia kuendelea kuenea kwa ugonjwa wa Usubi | | |
| **11.** | Huwa unafanya shughuli yoyote kati ya hizi karibu au kwenye mito?  (tick where appropriate) | 1. Kuogelea 2. Uvuvi 3. Kilimo 4. Kulisha wanyama(ng’ombe,mbuzi ,n.k) 5. Kuoga na kufua 6. Kuchota maji pekee 7. Hapana ,huwa sifanyi shughuli yoyte katika mito |
| **12.** | Una kawaida ya kwenda msituni? | 1. Ndiyo 2. Hapana |
| **13.** | Kama umejibu ndiyo kwenye swali la 21, huwa uanafanya shughuli gani msituni?  (weka alama ya vyema kwenye sehemu husika) | 1. Kuwinda 2. Kuokota matunda 3. Kucheza 4. Mengine |
| **14.** | Kama umejibu D. Mengine kwenye swali la 22 , tafadhali ainisha baadhi ya hizo shughuli |  |
| **15.** | Huwa unakunywa dawa za Usubi/ ivermectin? | 1. Ndiyo 2. Hapana |
| **16.** | Kama huwa haunywi dawa za usubi/ivermectin,ni kwasababu gani? | 1. Madhara yatokanayo na dawa kama ,kuwashwa na kuvimba mwili 2. Umbali wa kutembea kufuata dawa 3. Makatazo ya wazazi 4. Sijui chcochote kuhusu unywaji wa dawa za Usubi/Ivermectin |
| **17.** | Huwa unachukua tahadhari yoyote dhidi ya kung’atwa na wadudu? | 1. Kuvaa nguo ndefu,zenye mikono mirefu 2. Kupaka mafuta ya kuzuia wadudu 3. Mengine 4. Huwa sichukui tahadhari yoyote |
| **18.** | Kama umejibu C.Mengine kwenye swali la 25, unaweza kutaja baadhi ya njia unazotumia kujikinga dhidi ya kung’atwa na wadudu wakati wa mchana |  |
| **19.** | Huwa unatolea wapi maji ya kutumia? | 1. Bombani 2. Visima 3. Mito |
| **20.** | Matokeo ya maabara (OV16) | Anao  Hana |
